# Overcoming Barriers to Exclusive Breastfeeding in Lao PDR: Social Transfer Intervention Randomised Controlled Trial

**DOI:** 10.3390/nu17152396

**Published:** 2025-07-22

**Authors:** Najmeh Karimian-Marnani, Elizabeth Tilley, Jordyn T. Wallenborn

**Affiliations:** 1 Department of Epidemiology and Public Health, Swiss Tropical and Public Health Institute, Kreuzstrasse 2, 4123 Allschwil, Switzerland; 2 Department of Mechanical and Process Engineering, Swiss Federal Institute of Technology (ETH) Zurich, Rämistrasse 101, 8092 Zürich, Switzerland; tilleye@ethz.ch; 3 University of Basel, Petersplatz 1, 4001 Basel, Switzerland

**Keywords:** breastfeeding, exclusive breastfeeding, maternal health, financial incentives, infant nutrition, factors

## Abstract

**Background/Objectives**: Despite the numerous health benefits of exclusive breastfeeding (EBF) for the infant and the mother, EBF rates in Lao People’s Democratic Republic, Lao PDR, remain at 40%. We investigate how barriers to EBF were overcome by a social transfer intervention. **Methods**: Data from the Social Transfers for Exclusive Breastfeeding ongoing randomised controlled trial (RCT) (*n* = 298) in Vientiane, Lao PDR, was used. Mothers in the RCT were randomised equally into a control group, an unconditional transfer group and a conditional transfer group and followed up to six months (*n* = 280). We used logistic and Cox proportional hazards regression models to investigate the association of predictor variables with EBF at six months and the risk of EBF cessation in each of the three groups. **Results**: Greater breastfeeding self-efficacy increased the odds of EBF at six months in both intervention groups (unconditional transfer OR 1.39 [95% CI 1.09, 1.87, *p* = 0.02] and conditional transfer OR 1.26 [1.01, 1.61, *p* = 0.05]) and reduced the risk of EBF cessation (unconditional transfer HR 0.87 [0.77, 0.98, *p* = 0.02]). Maternal secondary and tertiary education in the intervention groups showed higher odds of EBF at six months and lower EBF cessation risk. Participants in the intervention group that intended to exclusively breastfeed in pregnancy showed a reduced EBF cessation risk in both intervention groups. **Conclusions**: Social transfers helped overcome the exclusive breastfeeding barrier of a higher education level and working status and improved EBF outcomes for mothers who intended to exclusively breastfeed and wanted the pregnancy. Breastfeeding self-efficacy positively influences EBF outcome, regardless of whether the mother received a social transfer or not.

## 1. Introduction

Breastmilk is optimally suited to an infant’s needs, containing essential vitamins and minerals, immunological components and microbiota [1,2,3]. Breastfeeding has been shown to protect against neonatal mortality, infections and digestive diseases shortly after birth [4,5], support cognitive development in infants [6] and reduce the long-term risk of obesity [7] and diabetes [8]. Mothers who breastfeed have a reduced risk of cardiovascular disease [9], breast and ovarian cancer [10] and postpartum depression [11].

The World Health Organisation (WHO) recommends initiating breastfeeding within the first hour of birth, exclusive breastfeeding (EBF) for the first six months and continuing breastfeeding (with additional food and drink) for 2 years and beyond. EBF is the practice of only feeding the infant with breastmilk and no other foods or liquids [12,13]. Despite WHO recommendations, the global EBF rate is only 44% and varies across different geographic regions [14].

Lao People’s Democratic Republic (Lao PDR) is a lower-middle income country in Southeast Asia with an EBF rate of 40% [15]. Each year, inappropriate breastfeeding practices deviating from WHO recommendations lead to 1597 infant deaths, 117 maternal deaths and 205,000 avoidable cases of childhood diarrhoea and pneumonia [16]. Treatment of maternal and infant avoidable conditions related to poor breastfeeding practices, amount to USD 312,000 in health system costs [16]. Total economic losses from inadequate breastfeeding amount to USD 235 million or 1.96% of Lao PDR’s gross national income: the highest loss in the region [16].

Understanding the facilitators and barriers to EBF in Lao PDR aids in supporting mothers to adopt correct breastfeeding practices. Sociocultural influences (e.g., local traditions), healthcare provisions (e.g., lactation counselling or antenatal visits), past birthing or breastfeeding experience and postpartum maternal factors (e.g., stress, anxiety) influence breastfeeding [17,18,19]. Mothers with higher breastfeeding self-efficacy (her level of confidence in successfully feeding her infant) and higher breastfeeding satisfaction are more likely to exclusively breastfeed for six months [20]. Sociodemographic (e.g., maternal age, education level) and socioeconomic factors (e.g., household wealth, employment) have additionally been shown to affect breastfeeding practices [21]. Younger maternal age, an earlier return to employment and higher wealth-index are some factors associated with lower likelihood of EBF up to six months [22,23,24,25,26].

Postpartum maternal traditions such as strict periods of confinement, dietary re-strictions, visits to steam rooms, steam baths, hot beds and mother roasting (i.e., the mother lies on a bed placed above charcoal embers) are widely practised in Lao PDR [27,28,29]. Cultural practices can create harsher postpartum conditions for the mother and could therefore negatively impact EBF rates [30]. The period of confinement ranges from 10 to 45 days, thought to promote postpartum healing through staying indoors in the warmth [30]. Strict postpartum diets, followed by more than 80% of new mothers in Lao PDR, restrict access to meat and other nutritious foods that provide the energy required for breastfeeding and the production of breastmilk [28]. No research has investigated the impact of these cultural factors on EBF rates.

Appropriate breastfeeding interventions can overcome the barriers to EBF. Providing participants with cash transfers as financial incentives has proven to be a successful approach in increasing breastfeeding rates and breastfeeding duration [31,32,33]. A recent randomised controlled trial conducted in Lao PDR is the first to explore the impact of financial incentives and social transfers in any low- or middle-income country (LMIC) [34,35]. Findings from the Social Transfer for Exclusive Breastfeeding randomised controlled trial demonstrated the efficacy of unconditional and conditional social transfers in improving EBF rates [34].

In this study, we aimed to (1) identify predictors of early cessation of EBF in Lao PDR and (2) to investigate whether EBF barriers may be mitigated with a social transfer intervention. We used data from the Social Transfer for Exclusive Breastfeeding pilot randomised controlled trial conducted in Vientiane, Lao PDR. We hypothesise that the intervention will reduce the impact of EBF barriers on EBF rates at six months and on cessation of EBF. Our findings aim to inform public health officials on how social transfer interventions can improve breastfeeding practices.

## 2. Materials and Methods

### 2.1. Data Source

We used data from the Vientiane Multi-Generational Birth Cohort (VITERBI) study and the Social Transfer for Exclusive Breastfeeding (STEB) randomised controlled trial (RCT) [34,35,36]. VITERBI follows 3000 recruited pregnant mothers and is the first multi-generational longitudinal birth cohort in Lao PDR. Nested within VITERBI, STEB is an RCT aiming to assess the effect of a social transfer intervention on EBF rates in Lao PDR, running from August 2022 to December 2026. Both STEB and VITERBI were conducted in four districts in the capital city, Vientiane: Chanthabuly, Sangthong, Sikhottabong and Parkngum. The former two districts represent higher-income areas (urban), while the latter two represent lower-income regions (rural).

Pregnant women from VITERBI were screened for eligibility for STEB during a phone interview to ensure they met the following criteria: had an expected due date/gave birth between July 1 2022 and June 30 2023; gave birth within the last four weeks prior to inclusion start; were exclusively breastfeeding at the time of recruitment; had no illnesses that contraindicated breastfeeding; and had a healthy singleton infant of 37 weeks or more gestation with a birth weight of at least 2500 g.

STEB participants (*n* = 298) were randomly assigned to one of three groups: a control group, receiving breastfeeding education only; an unconditional social transfer group, receiving a social transfer at six months postpartum; and a conditional social transfer group receiving a social transfer at six months postpartum on the condition that they are still exclusively breastfeeding. All participants received identical educational pamphlets on the benefits of exclusive breastfeeding and tips to overcome insufficient milk supply. Randomisation allocation ratio was 1:1:1 and occurred using a random number draw generated by the Open Data Kit (ODK) software (version 2023021307) for data capture on tablets.

Mothers were interviewed at one month postpartum between July 2022 and April 2023 and again at six months postpartum between December 2022 and October 2023. They answered questions on labour and delivery, maternal and child health measurements and infant feeding. Biospecimen samples (e.g., blood sample or breast milk samples) were also collected. Until the end of the visit at one month postpartum, neither the research staff nor participants were aware of outcome of randomization. After this time, blinding of investigators, research staff and participants was not feasible.

**Figure 1:**
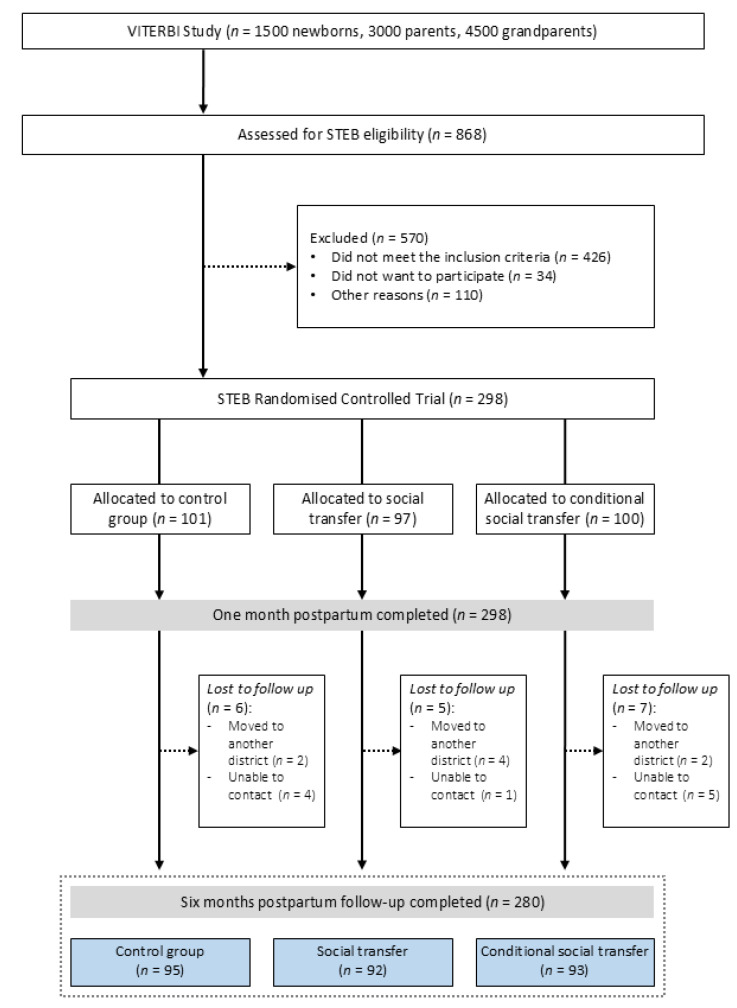
Flowchart of participant’s disposition throughout the study. Participants eligible for the Social Transfers for Exclusive Breastfeeding (STEB) randomised controlled trial were recruited from the Vientiane Multi-Generational Birth Cohort (VITERBI). A total of 298 participants were divided into three groups: a control group, unconditional social transfer group and conditional social transfer group.

The social transfer included either (1) cash, (2) diapers, (3) baby clothes or (4) development toys, as selected by the participants themselves. Each option could be chosen individually or as a combination, as long as it did not exceed the pre-determined value of USD 75 per participant. Eighteen participants were lost to follow-up at six months, giving a total of *n* = 280 (Figure 1). Attrition was distributed as follows: 6 participants from the control group, 5 from the unconditional social transfer group and 7 from the conditional transfer group. As the intervention was delivered at the six-month visit, those lost to follow-up prior to this time point did not receive the intervention or provide outcome data. A complete case analysis was conducted, so those participants were excluded from the analysis.

### 2.2. Exclusive Breastfeeding: Outcome Variable

**Figure 2:**
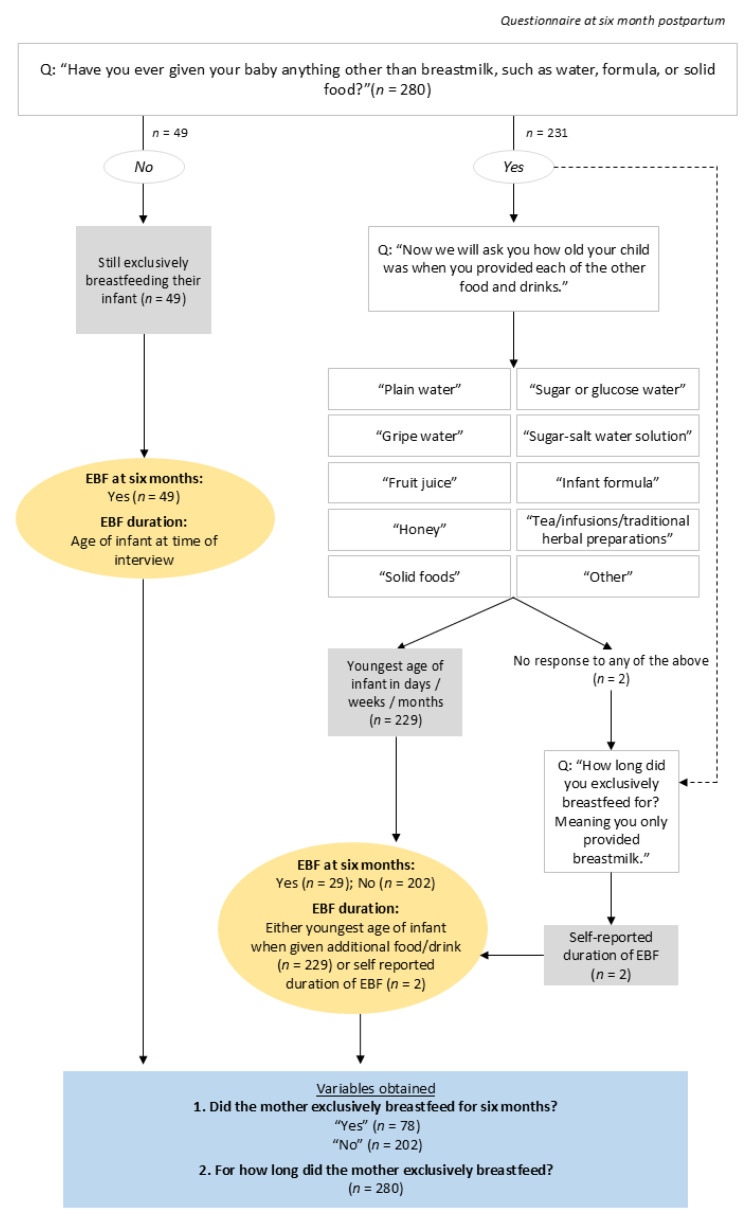
Exclusive breastfeeding at six months and exclusive breastfeeding duration as desired outcomes. Those who were still exclusively breastfeeding at six months were automatically categorised as such. Exclusive breastfeeding status at six months and exclusive breastfeeding duration of those not exclusively breastfeeding at the time of the six-month postpartum survey were calculated by the age of the infant when additional foods or drinks were given. Where the response to this survey question remained empty (*n* = 2), the self-reported duration of exclusive breastfeeding was used.

The primary outcome of interest, EBF, was determined from participant responses to questions from the six months postpartum questionnaire. The mother was asked whether she was still breastfeeding, had already introduced complementary foods or drinks (and if so when) and her duration of EBF (Figure 2). If at the time of the six-month survey the mother reported herself to be exclusively breastfeeding, her duration of EBF was calculated as the number of days difference between giving birth and the date of interview, with her EBF status at six months categorised as “Yes”. If the mother was no longer exclusively breastfeeding at six months, we computed the number of days difference between the birth date and when EBF stopped (i.e., age when the infant was introduced to complementary food or drink). Where the earliest date of receiving additional food or drink was missing, participant responses to the question of their duration of EBF was used. Samples of 50 mL of human milk collected at six months postpartum served to confirm self-reported declarations of continued breastfeeding to ensure accuracy of breastfeeding responses.

### 2.3. Independent Variables

Predictor variables were allocated into five categories for ease of understanding and to reduce complexity: sociodemographic and economic, maternal intention and experience, maternal satisfaction, maternal mental health and cultural practices. A figure in the appendix illustrates this (Appendix A.1).

Sociodemographic and economic variables included participants’ age, education, marital status, employment status at six months postpartum and household wealth index. Age was categorised into three groups: younger than 25, between 25 and 30 and older than 30. Education categories included primary education and no schooling together, secondary education as a second group and tertiary education or higher as a third group. Marital status described women who were married or cohabitating in one and those who were not married in another category. Employment status was recorded as the working status of the participant at six months postpartum; the “not working” category included women on maternity leave, unemployed women, homemakers or any other reason. Household asset possession was classed into quintiles using principal component analysis, as detailed in the Demographic Health Survey Wealth Index [37]. Variables with factor loadings of <0.2, or with no heterogeneity in responses, were removed from the index analysis.

The second category of maternal experience and intention encompassed previous live births (first birth; previously given birth), feeding intention at pregnancy (EBF; with additional foods) and whether the mother wanted to be pregnant at the time (yes; no).

Maternal satisfaction included the Breastfeeding Self-Efficacy Scale-Short Form (BSES-SF) and engagement of the mother in caregiver activities for development [38,39]. The BSES-SF score was derived using factor loadings from the original scale, giving total scores ranging from 14 to 70, where a higher score indicates a higher breastfeeding self-efficacy. A principal component analysis of questions related to caregiver activities identified factor loadings for scoring, ranging from 8 to 40, with a lower score indicating a higher frequency of caregiving activities.

Maternal mental health was evaluated using the Postpartum Specific Anxiety Scale (PSAS-RSF-C), the Perceived Stress Scale (PSS) and the Grit Scale [40,41,42]. Previously identified factor loadings of the PSAS-RSF-C questionnaire were used to score participants’ responses from 12 to 48. Having postpartum anxiety was categorised as having a score of 26 or greater [42]. The PSS scoring was determined from used factor loadings from a study on Vietnamese women, to use a culturally similar cohort [43]. Scores ranging from 0 to 13 were considered low stress; 14–26 was moderate stress and 27–40 was high stress (though no participants fell into the latter category) [44]. The Grit score of each participant was calculated based on the mean of the individual Grit Scale questions, ranging from 1 to 5 representing not at all gritty to extremely gritty, respectively.

Cultural practices (yes; no) included participation in at least one of the following: hotbeds, mother roasting or steam saunas. No participants engaged in steam baths. Duration of confinement (in days) was also measured as a continuous scale. Those that were not confined were set to have a duration of “0”.

### 2.4. Confounding Variables

Though any confounding factors would be minimised through randomisation, we adjusted for confounders to address group imbalances, given the small sample size of the trial groups [45]. We identified potential confounding factors that may impact our analysis through a literature search [46,47,48,49] and then assessed their frequencies. Confounders we considered important to adjust for include the following: (1) if the infant had any illness requiring a doctor’s visit since birth—could impact medical advice related to breastfeeding; (2) if the mother was taking medication during the time she was breastfeeding—which can impact breastfeeding continuation if the mother is on certain medication; (3) how many days postpartum she was—accounting for differences in postpartum time period during the interview dates and (4) her number of antenatal visits during pregnancy—a proxy for maternal engagement with health services and exposure to breastfeeding counselling. Any one, a combination, or all of these confounders were adjusted for in models, where they showed a >10% regression coefficient difference with the independent variables. This was done to ensure greater precision and confidence in the impact of the independent variables.

### 2.5. Statistical Analysis

Descriptive statistics was used to obtain frequencies and percentages for categorical variables, and mean and standard deviations (SDs) were used for continuous variables. Pearson’s Chi-squared test was used to identify significant differences between the control and treatment groups.

Logistic regression models estimated the association of predictor variables with EBF status at six months using odds ratios. Cox proportional hazards regression models displayed the risk of EBF cessation using hazard ratios. Given the exploratory nature of this study, as well as the demonstrated impact of the intervention, we stratified all models by trial group (control group; unconditional social transfer and conditional social transfer).

Initially, unadjusted regression models for each predictor variable were run. Following the 10% coefficient difference rule for confounders, parsimonious regression models of the crude predictor with the statistically significant confounder were run. Then, fully adjusted (logistic and Cox proportional hazards) for all predictor variables and the confounders (for each RCT arm) were run. When variables were homogeneous (i.e., no variability in answers) or had few participants in each category, the variable was not added in the fully adjusted model. Given the small sample size across each RCT arm, *p* < 0.1 was used to demonstrate statistical significance [50].

Data analysis was performed using R statistical computing environment, using “tidyverse” for data manipulation and the “ggplot2” package for data visualisation [51,52]. “Survival” and “survminer” packages were used specifically for the Cox proportional hazards regression models [53,54].

## 3. Results

### 3.1. Characteristics of the Participants

Of 298 participants enrolled, 101 were randomised into the control group, 97 into the unconditional social transfer group and 100 into the conditional social transfer group. Eighteen participants were lost to follow-up or had missing data (*n* = 280) (Figure 1). Table 1 presents the descriptive statistics: mean age of participants is 27.2; the majority are married or cohabitating (93%), received secondary education (40%) and were not working (82% at six months postpartum). Most participants have previously given birth (61%) and intended to exclusively breastfeed their baby (86%). Almost all mothers wanted the pregnancy (95%). Mother roasting (90%) and confinement (95%) were the most common postpartum traditions. Over a quarter (28%) of all participants were exclusively breastfeeding at six months. A statistically significant difference between the three RCT arms is observed in the employment status of the participants at six months, the household wealth index, intention to feed and the outcome of EBF at six months. Other variables show no statistical difference between the three trial arms.

### 3.2. Association with Exclusive Breastfeeding Status at Six Months

Table 2 displays odds ratios from logistic regression models for the three RCT arms, depicting the association of predictor variables with EBF at six months. At six months postpartum, the control group showed an 80% decrease in EBF if the mother had secondary education (OR = 0.2; 95% CI 0.03, 1.3; *p* = 0.09) compared with those with primary education or no schooling, though only marginally significant. Other variables did not show an association with EBF in this group.

In the unconditional social transfer group, an increase by one score of breastfeeding self-efficacy significantly increased odds of EBF at six months by 1.39 (95% CI 1.09, 1.87; *p* = 0.02). There was no significant association between EBF status and any other variables in this group.

In the conditional transfer group, a higher breastfeeding self-efficacy score (OR = 1.26; 95% CI 1.01, 1.61; *p* = 0.05) and not having postpartum anxiety (OR = 0.29; 95% CI 0.07, 1.12; *p* = 0.08) increased the odds of EBF at six months postpartum, with marginal statistical significance. A longer confinement duration in the conditional transfer group showed a marginally significant negative association with EBF status at six months (OR = 0.97; 95% CI 0.94, 1.00; *p* = 0.06).

### 3.3. Risk of Exclusive Breastfeeding Cessation

Table 3 presents hazard ratios from the Cox proportional hazards model, depicting the risk of EBF cessation for the predictor variables. Having secondary education in the control group increased the risk of EBF cessation compared with primary education (HR = 2.07; 95% CI 0.95, 4.48; *p* = 0.07) yet with marginal significance. An increase in breastfeeding self-efficacy score showed marginal significance in reducing EBF discontinuation risk by 11% (HR = 0.89; 95% CI 0.78, 1.01; *p* = 0.08) in the control group.

Going to work significantly increased EBF cessation risk by more than double (HR = 2.32; 95% CI 1.06, 5.09; *p* = 0.04) in the unconditional transfer group. A higher breastfeeding self-efficacy reduced EBF by 13% in this group (HR = 0.87; 95% CI 0.77, 0.98; *p* = 0.02). A higher grit score in the unconditional transfer group reduced the risk of EBF discontinuation by 51% (HR = 0.51; 95% CI 0.25, 1.05; *p* = 0.07) and with marginal significance. A prolonged duration of confinement showed a marginal association with EBF status (HR = 0.99; 95% CI 0.97, 1.00; *p* = 0.10).

No variables in the conditional social transfer group showed a significant association with EBF.

Table A1 presents unadjusted odds ratios and parsimonious model odds ratios of the association between the independent variables and EBF at six months (Appendix A.2). The results are in line with that of Table 2, with an additional depiction of the suggested association between a higher grit score and EBF status at six months (crude OR = 3.69; 95% CI 1.00, 15.00; *p* = 0.06). Similarly, Table A2 depicts hazard ratios for EBF cessation risk for the independent variables at any given point in time using both unadjusted and parsimonious models (Appendix A.3). Desire for the pregnancy shows a statistically significant decreased risk of EBF cessation, in line with findings of Table 3 (adjusted HR = 0.42; 95% CI 0.19, 0.92; *p* = 0.03).

## 4. Discussion

Early cessation of EBF carries negative health outcomes for the mother and infant. We investigated the impact of a social transfer intervention in Lao PDR on barriers to EBF for women. This intervention is the first of its kind in Southeast Asia and in any LMIC. Our findings highlight the strong associations between breastfeeding self-efficacy, working at six months, maternal education, social status and EBF. The social transfer intervention was shown to overcome differences in maternal education, household wealth and breastfeeding self-efficacy scores. Cultural traditions did not show a significant effect on EBF rates.

Having secondary education in the control group, compared with no schooling or primary education, increased the risk of EBF cessation. In the intervention groups, less of a difference was seen between the different educational levels. Maternal education can have a positive effect on EBF, as mothers are better able to make informed decisions on infant nutrition [55,56]. However, women with secondary or tertiary education in LMICs reported lower EBF rates compared to those with lower schooling [57,58]. Higher levels of education are positively correlated with socioeconomic status, formula milk purchasing and being occupationally busy, thus spending periods of the day away from their infant [59,60,61]. Thus, the social transfer intervention may have helped mothers to overcome the barrier of having a higher educational background.

Employed mothers are less likely to continue exclusively breastfeeding due to the time commitments working requires: being away from the infant, as well as the emotional and physical stress that would hinder breastfeeding [62,63,64,65]. Our study confirms such findings, as we found that mothers who were working at six months showed a higher risk of EBF cessation. It is plausible that the conditional social transfer intervention overcame maternal working status as an EBF barrier of employment by providing more financial support for breastfeeding, in line with other cash transfer studies [31,32,33], though this would need further studies with larger sample sizes to confirm.

The higher the mother’s breastfeeding self-efficacy, the higher her confidence in performing the task, and therefore the lower the likelihood of EBF discontinuation [66,67,68]. Consistent with previous research, our findings show that a higher self-efficacy score gave increased odds of still exclusively breastfeeding at six months across all groups and a lower risk of EBF cessation at any given time point. The unconditional social transfer showed the strongest effect of self-efficacy on EBF rate. Efforts to increase self-efficacy have been shown to be a strong enough intervention on their own to increase EBF rates, consistent with our findings where all groups showed positive EBF outcomes with a higher score [69,70,71,72]. Given that inter-group differences were not assessed in this study, it is difficult to determine whether self-efficacy was improved with the educational material or the intervention, as EBF cessation risk was reduced across all groups. Nonetheless, the intervention proved to significantly extend breastfeeding duration in women with greater confidence in breastfeeding.

Prenatal breastfeeding intention is a strong predictor of EBF duration [73,74,75,76]. Mothers who intend to exclusively breastfeed are more aware of the benefits of EBF and seek more sources of information on infant nutrition [77]. Other factors such as maternal education level, age, being multiparous and the pregnancy trimester influence breastfeeding intention [78,79]. Findings from our supplementary analysis show that exclusive breastfeeding intention in the conditional social transfer group reduced EBF cessation risk. Despite our primary findings showing no association between EBF intention and EBF, patterns from the hazard ratios show that the intervention groups could be presenting a lower EBF cessation risk, though these are not conclusive nor significant; further studies would be required to support this. It is possible that the intervention drew attention to EBF and motivated mothers to realise their goal of EBF.

Intending for the pregnancy, pregnancy intention, motivates mothers to continue exclusively breastfeeding, due to their desire for being a mother and knowing what to expect [80,81,82]. Our primary findings did not show any significant association between pregnancy intention and EBF status. However, additional parsimonious and unadjusted regression models showed a reduction in EBF cessation risk in mothers who wanted the pregnancy and who were assigned to the unconditional social transfer group. The unconditional social transfer may have offered support to the mothers that wanted the pregnancy in continuing EBF, without the added pressure of the transfer being conditional.

As a pilot study, this study was disadvantaged with a small number of participants. This meant that some analyses could not be conducted due to homogeneity in the variable responses, and in subgroup analyses that included fewer participants in each group. The wide confidence intervals obtained as a result demonstrated imprecision in some of the findings. A larger study sample would be necessary, especially to accurately compare between the groups. Given that STEB was a pilot RCT, such results are not expected for future social transfer interventions that recruit a larger number of participants. Furthermore, despite the study taking place in both urban and rural districts of Vientiane, findings may not be completely relevant to rural areas in Lao PDR outside of the capital.

This study is sensitive to recall bias in participants remembering how long they exclusively breastfed for. Efforts to overcome this were made by specifically asking when they fed their infant with different foods or drinks. Nonetheless, breastfeeding research shows that maternal recall of infant feeding and lactation is typically reliable [83]. Social desirability bias further presents in breastfeeding research, i.e., when the mother provides a longer duration of exclusive breastfeeding for fear of judgement or to receive the social transfer. To control for this, the research team employed techniques such as breastmilk sampling and speaking with family members about when additional foods or drinks were given.

We did not conduct an a priori sample size calculation for this study, rather relying on the sample size calculation of the original STEB study [34]. It would be important for future research to conduct such calculations before data collection to enhance validity and generalisability of the results [84]. Moreover, our analyses statistically examined multiple predictors across three trial arms—increasing the potential for Type I error due to multiple comparisons. As this was an exploratory analysis, we did not apply formal corrections (e.g., Bonferroni) and results should therefore be interpreted with caution [85,86].

## 5. Conclusions

The social transfer intervention helped overcome the EBF barrier of a higher education level and working status of the mother at six months. The intervention further supported women who intended on exclusively breastfeeding their infant and wanted the pregnancy to achieve their goal of EBF. We further identified that breastfeeding self-efficacy positively influences EBF outcomes, regardless of whether the mother received an intervention or not. Larger studies are needed to more accurately compare control and intervention group differences for each factor, in order to determine to what extent EBF barriers were overcome. Future work would involve a larger study cohort and geographical expansion beyond Vientiane for more accurate representation of the population.

## Figures and Tables

**Table 1:** Descriptive statistics of the participants at baseline.

Characteristic	Overall (*n* = 298)	Randomised Controlled Trial Arm			*p*-Value ^1^
		Control (*n* = 101)	Unconditional Social Transfer (*n* = 97)	Conditional Social Transfer (*n* = 100)	
	***n* (%)**				
Maternal age (years)					0.12
Younger than 25	92 (31.2%)	32 (32.0%)	33 (34.4%)	27 (27.3%)	
Between 25 and 30	104 (35.3%)	32 (32.0%)	27 (28.1%)	45 (45.5%)	
Older than 30	99 (33.6%)	36 (36.0%)	36 (37.5%)	27 (27.3%)	
Marital status					0.50
Married or cohabitating	274 (92.9%)	93 (93.0%)	87 (90.6%)	94 (94.9%)	
Not married	6 (7.1%)	8 (7.0%)	10 (9.4%)	6 (5.1%)	
Maternal education					0.20
Primary or no education	76 (25.8%)	21 (21.0%)	25 (26.0%)	30 (30.0%)	
Secondary	119 (40.3%)	43 (43.0%)	44 (45.8%)	32 (32.3%)	
Tertiary	100 (33.9%)	36 (36.0%)	27 (28.1%)	37 (37.4%)	
District in Vientiane					0.40
Chanthabuly	31 (10.9%)	9 (9.2%)	11 (12.1%)	11 (11.5%)	
Pakngum	67 (23.5%)	17 (17.3%)	23 (25.3%)	27 (28.1%)	
Sangthong	64 (22.5%)	23 (23.5%)	24 (26.4%)	17 (17.7%)	
Sikhottabong	123 (43.2%)	49 (50.0%)	33 (36.3%)	41 (42.7%)	
Employment status at one month					0.20
Working	8 (2.8%)	1 (1.0%)	2 (2.2%)	5 (5.4%)	
Not working	274 (97.2%)	96 (99.0%)	90 (97.8%)	88 (94.6%)	
Household wealth index					0.02
1st quartile	63 (21.1%)	15 (14.9%)	27 (27.8%)	21 (21.0%)	
2nd quartile	57 (19.1%)	12 (11.9%)	25 (25.8%)	20 (20.0%)	
3rd quartile	59 (19.8%)	28 (27.7%)	12 (12%)	19 (19.0%)	
4th quartile	80 (26.8%)	29 (28.7%)	25 (26%)	26 (26.0%)	
5th quartile	39 (13.1%)	17 (16.8%)	8 (8.2%)	14 (14.0%)	
Previous births					0.50
No previous births	115 (39.1%)	35 (35.0%)	37 (38.9%)	43 (43.4%)	
At least one previous birth	179 (60.9%)	65 (65.0%)	58 (61.1%)	56 (56.6%)	
Intention to feed the baby					0.01
Exclusive breastfeeding	254 (86.1%)	78 (78.0%)	85 (88.5%)	91 (91.9%)	
Complementary feeding	41 (13.9%)	22 (22.0%)	11 (11.5%)	8 (8.1%)	
Desire for the pregnancy					0.60
Wanted the pregnancy	279 (94.6%)	96 (96.0%)	89 (92.7%)	94 (94.9%)	
Did not want the pregnancy	16 (5.4%)	4 (4%)	7 (7.3%)	5 (5.1%)	

^1^ Pearson’s Chi-squared and Fisher’s exact tests were used to obtain *p*-values.

**Table 2:** Odds of exclusive breastfeeding at six months by predictor variables, using fully adjusted logistic regression.

Variable	Category	Randomised Controlled Trial Arm					
		Control		Unconditional Social Transfer		Conditional Social Transfer	
		OR (95% CI)	*p*	OR (95% CI)	*p*	OR (95% CI)	*p*
Age	Younger than 25	—	—	—	—	—	—
	Between 25 and 30	0.61 (0.09, 3.91)	0.60	1.86 (0.41, 9.38)	0.43	0.30 (0.06, 1.36)	0.13
	Older than 30	2.02 (0.36, 12.80)	0.43	1.11 (0.26, 5.14)	0.89	0.48 (0.09, 2.62)	0.40
Marital status	Not married	—	—	—	—	—	—
	Married or cohabitating	N/A	N/A	1.15 (0.11, 15.60)	0.91	N/A	N/A
Maternal education	Primary or no education	—	—	—	—	—	—
	Secondary	0.20 * (0.03, 1.30)	0.09	0.72 (0.16, 3.27)	0.66	1.03 (0.19, 5.53)	0.97
	Tertiary	0.33 (0.04, 2.51)	0.29	1.64 (0.30, 9.14)	0.56	0.85 (0.15, 4.83)	0.85
Employment status at six months	Not working	—	—	—	—	—	—
	Working	N/A	N/A	N/A	N/A	0.20 (0.01, 1.65)	0.18
Household wealth ^1^		1.25 (0.67, 2.52)	0.51	1.08 (0.66, 1.77)	0.76	1.54 (0.86, 2.96)	0.16
Previous births	Never given birth	—	—	—	—	—	—
	At least one previous birth	0.49 (0.11, 2.17)	0.34	1.20 (0.29, 5.27)	0.80	0.66 (0.19, 2.17)	0.49
Intention to feed the baby	Complementary feeding	—	—	—	—	—	—
	Exclusive breastfeeding	0.45 (0.08, 2.52)	0.35	0.48 (0.09, 2.64)	0.39	N/A	N/A
Desire for the pregnancy	Did not want the pregnancy	—	—	—	—	—	—
	Wanted the pregnancy	N/A	N/A	N/A	N/A	0.16(0.01, 1.80)	0.17
Breastfeeding self-efficacy ^1^		1.21 (0.86, 1.82)	0.30	1.39 ** (1.09, 1.87)	0.02	1.26 ** (1.01, 1.61)	0.05
Caregiver activities ^1^		0.85 (0.46, 1.50)	0.58	0.94 (0.61, 1.43)	0.77	0.87 (0.51, 1.46)	0.59
Postpartum anxiety	Has anxiety	—	—	—	—	—	—
	No postpartum anxiety	0.78 (0.15, 3.53)	0.75	1.94 (0.56, 7.41)	0.31	0.29 * (0.07, 1.12)	0.08
Perceived stress	Low stress	—	—	—	—	—	—
	Moderate stress	0.69 (0.15, 3.09)	0.62	1.41 (0.42, 5.05)	0.58	0.80 (0.23, 2.80)	0.73
Grit scale ^1^		N/A	N/A	N/A	N/A	1.32 (0.34, 5.10)	0.68
Participation in at least one of these: hotbed, mother roasting or steam sauna	Did not participate	—	—	—	—	—	—
	Participated in at least one	N/A	N/A	0.44 (0.07, 3.04)	0.38	0.91 (0.10, 9.26)	0.93
Confinement duration ^1^		1.00 (0.95, 1.05)	0.87	1.03 (0.99, 1.06)	0.13	0.97 * (0.94, 1.00)	0.06

^1^ Continuous variables with a scale or scoring system. OR—Odds ratio. CI—Confidence interval. Values with significant *p* values are in bold: * *p* < 0.1, ** *p* < 0.05. N/A—the variable was not included in the model due to homogeneity in the groups or a sample size < 8 per category. All models were adjusted for maternal medication, if the baby had an illness after birth and the number of days postpartum. Control and conditional social transfer groups were additionally adjusted for the number of antenatal visits.

**Table 3:** Risk of exclusive breastfeeding cessation over time by predictor variables, using fully adjusted Cox proportional hazards regression.

Variable	Category	Randomised Controlled Trial Arm					
		Control		Unconditional Social Transfer		Conditional Social Transfer	
		HR (95% CI)	*p*	HR (95% CI)	*p*	HR (95% CI)	*p*
Age	Younger than 25	—	—	—	—	—	—
	Between 25 and 30	1.03 (0.53, 2.01)	0.92	1.12 (0.57, 2.18)	0.75	1.73 (0.77, 3.92)	0.19
	Older than 30	0.61 (0.33, 1.10)	0.10	0.92 (0.48, 1.78)	0.81	1.52 (0.59, 3.90)	0.39
Marital status	Not married	—	—	—	—	—	—
	Married or cohabitating	1.97 (0.65, 5.99)	0.23	1.03 (0.36, 2.95)	0.96	2.08 (0.70, 6.15)	0.19
Maternal education	Primary or no education	—	—	—	—	—	—
	Secondary	2.07 * (0.95, 4.48)	0.07	1.11 (0.54, 2.29)	0.77	0.93 (0.42, 2.06)	0.86
	Tertiary	1.26 (0.54, 2.93)	0.60	0.78 (0.34, 1.78)	0.56	1.19 (0.53, 2.66)	0.67
Employment status at six months	Not working	—	—	—	—	—	—
	Working	1.43 (0.78, 2.63)	0.24	2.32 ** (1.06, 5.09)	0.04	0.95 (0.43, 2.11)	0.90
Household wealth ^1^		0.94 (0.76, 1.17)	0.60	1.13 (0.87, 1.48)	0.35	0.92 (0.71, 1.19)	0.55
Previous births	Never given birth	—	—	—	—	—	—
	At least one previous birth	1.51 (0.89, 2.56)	0.13	1.32 (0.64, 2.72)	0.44	1.08 (0.60, 1.94)	0.80
Intention to feed the baby	Complementary feeding	—	—	—	—	—	—
	Exclusive breastfeeding	1.22 (0.67, 2.21)	0.52	0.80 (0.29, 2.20)	0.66	0.88 (0.34, 2.30)	0.80
Desire for the pregnancy	Did not want the pregnancy	—	—	—	—	—	—
	Wanted the pregnancy	1.04 (0.29, 3.78)	0.95	0.47 (0.19, 1.16)	0.10	1.29 (0.44, 3.74)	0.64
Breastfeeding self-efficacy ^1^		0.89 * (0.78, 1.01)	0.08	0.87 ** (0.77, 0.98)	0.02	0.95 (0.86, 1.05)	0.30
Caregiver activities ^1^		1.06 (0.83, 1.35)	0.64	1.08 (0.87, 1.33)	0.50	1.18 (0.92, 1.50)	0.19
Postpartum anxiety	Has anxiety	—	—	—	—	—	—
	No postpartum anxiety	1.22 (0.70, 2.30)	0.43	1.16 (0.67, 2.00)	0.59	1.58 (0.85, 2.91)	0.15
Perceived stress	Low stress	—	—	—	—	—	—
	Moderate stress	1.13 (0.65, 1.97)	0.67	1.16 (0.69, 1.93)	0.58	0.97 (0.54, 1.73)	0.91
Grit scale ^1^		0.52 (0.21, 1.29)	0.16	0.51 * (0.25, 1.05)	0.07	1.04 (0.56, 1.92)	0.91
Participation in at least one of these: hotbed, mother roasting or steam sauna	Did not participate	—	—	—	—	—	—
	Participated in at least one	1.00 (0.41, 2.44)	0.99	1.51 (0.59, 3.88)	0.39	0.90 (0.35, 2.35)	0.83
Confinement duration ^1^		1.00 (0.99, 1.02)	0.87	0.99 * (0.97, 1.00)	0.10	1.00 (0.99, 1.02)	0.53

^1^ Continuous variables with a scale or scoring system. HR—Hazard ratio. CI—Confidence interval. Values with significant *p* values are in bold: * *p* < 0.1, ** *p* < 0.05. The three models were adjusted for maternal medication, if the baby had an illness after birth and the number of antenatal visits during pregnancy.

**Figure A1:**
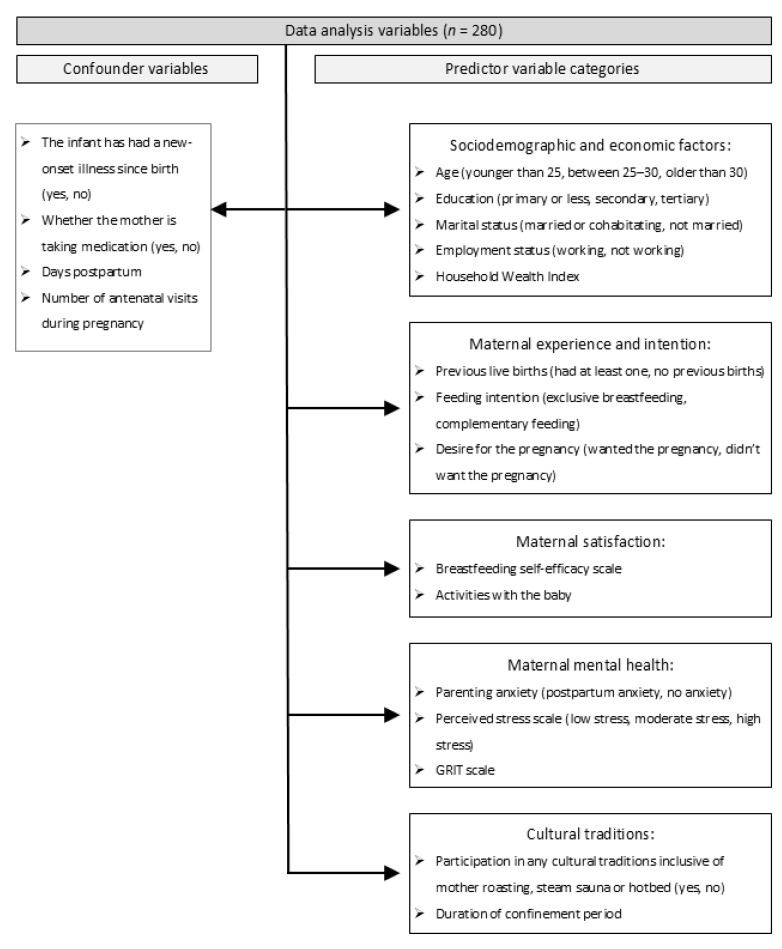
Categories of independent and confounder variables for the data analysis. On the right, predictor variables are broken down into five assigned categories based on their theme. All confounding variables are presented to the left—though different combinations are adjusted for in the models. Categorical variables are followed by their response options in brackets. Continuous variables are left as is.

## Data Availability

Data described in the manuscript, codebook and analytic code will be made available upon request, pending [e.g., application and approval, payment, other].

## Abbreviations

The following abbreviations are used in this manuscript:

CI	Confidence interval
EBF	Exclusive breastfeeding
HR	Hazard ratio
Lao PDR	Lao People’s Democratic Republic
LMIC	Low- or middle-income country
OR	Odds ratio
RCT	Randomised controlled trial
STEB	Social Transfers for Exclusive Breastfeeding
VITERBI	Vientiane Multi-Generational Birth Cohort

## Appendix A

### Appendix A.2. Unadjusted and Adjusted Logistic Regression Models

**Table A1:** Odds of exclusive breastfeeding at six months, by predictor variable and trial arm, using unadjusted and adjusted logistic regression models.

Variable	Category	Randomised Controlled Trial Arm											
		Control				Unconditional Social Transfer				Conditional Social Transfer			
		Unadjusted		Adjusted		Unadjusted		Adjusted		Unadjusted		Adjusted	
		cOR (95% CI)	*p*	aOR (95% CI)	*p*	cOR (95% CI)	*p*	aOR (95% CI)	*p*	cOR (95% CI)	*p*	aOR (95% CI)	*p*
Age ^A^	Younger than 25	—	—	—	—	—	—	—	—	—	—	—	—
	Between 25 and 30	0.69 (0.13, 3.45)	0.65	0.79 (0.14, 4.10)	0.78	1.11 (0.34, 3.57)	0.87	1.02 (0.31, 3.35)	0.98	0.70 (0.25, 1.94)	0.49	0.59 (0.20, 1.73)	0.34
	Older than 30	1.56 (0.42, 6.56)	0.51	1.69 (0.44, 7.39)	0.46	0.91 (0.30, 2.81)	0.87	0.82 (0.26, 2.61)	0.74	0.93 (0.30, 2.88)	0.90	0.75 (0.22, 2.47)	0.64
Marital status	Not married	—	—	—	—	—	—	—	—	—	—	—	—
	Married or cohabitating	0.87 (0.13, 17.30)	0.90	No adj		1.36 (0.30, 9.61)	0.71	No adj		N/A	N/A	N/A	N/A
Maternal Education ^B^	Primary or no education	—	—	—	—	—	—	—	—	—	—	—	—
	Secondary	0.46 (0.10, 2.15)	0.31	0.39 (0.08, 1.92)	0.24	0.70 (0.22, 2.26)	0.54	0.70 (0.21, 2.35)	0.81	1.02 (0.36, 2.91)	0.97	1.14 (0.38, 3.44)	0.81
	Tertiary	0.83 (0.21, 3.64)	0.79	0.68 (0.15, 3.17)	0.61	1.54 (0.47, 5.29)	0.48	1.40 (0.41, 4.96)	0.56	0.83 (0.30, 2.30)	0.71	0.90 (0.30, 2.76)	0.86
Employment status at six months	Not working	—	—	—	—	—	—	—	—	—	—	—	—
	Working	0.41 (0.06, 1.66)	0.27	No adj		0.16 * (0.01, 0.88)	0.09	No adj		0.34 (0.05, 1.47)	0.19	No adj	
Household wealth ^1,C^		1.18 (0.76, 1.91)	0.48	1.19 (0.76, 1.95)	0.47	1.11 (0.78, 1.57)	0.57	1.08 (0.75,1.54)	0.68	1.13 (0.83, 1.54)	0.44	1.20 (0.87, 1.69)	0.27
Previous births ^C^	Never given birth	—	—	—	—	—	—	—	—	—	—	—	—
	At least one previous birth	0.68 (0.21, 2.25)	0.51	0.76 (0.23, 2.60)	0.65	0.86 (0.34, 2.25)	0.75	0.93 (0.36, 2.50)	0.89	0.99 (0.43, 2.29)	0.98	1.03 (0.44, 2.43)	0.95
Intention to feed the baby ^C^	Complementary feeding	—	—	—	—	—	—	—	—	—	—	—	—
	Exclusive breastfeeding	0.67 (0.20, 2.70)	0.55	0.65 (0.19, 2.61)	0.51	0.43 (0.10, 1.88)	0.24	0.37 (0.09, 1.67)	0.18	4.63 (0.75, 89.40)	0.16	3.72 (0.58, 72.80)	0.24
Desire for the pregnancy ^C^	Did not want the pregnancy	—	—	—	—	—	—	—	—	—	—	—	—
	Wanted the pregnancy	N/A	N/A	N/A	N/A	N/A	N/A	N/A	N/A	0.16 (0.01, 1.14)	0.11	0.19 (0.01, 1.39)	0.15
Breastfeeding self-efficacy ^1^		1.12 (0.87, 1.46)	0.36	No adj	No adj	1.31 ** (1.06, 1.63)	0.01	No adj	No adj	1.07 (0.93, 1.24)	0.34	No adj	No adj
Caregiver activities ^1^		0.75 (0.46,1.20)	0.24	No adj	No adj	0.88 (0.61, 1.23)	0.46	No adj	No adj	0.81 (0.57, 1.15)	0.25	No adj	No adj
Postpartum Anxiety ^C^	Has anxiety	—	—	—	—	—	—	—	—	—	—	—	—
	No postpartum anxiety	1.05 (0.30, 3.35)	0.93	0.96 (0.26, 3.18)	0.95	2.08 (0.79, 5.97)	0.15	2.04 (0.77, 5.90)	0.17	0.60 (0.26, 1.39)	0.24	0.59 (0.25, 1.39)	0.23
Perceived stress ^C^	Low stress	—	—	—	—	—	—	—	—	—	—	—	—
	Moderate stress	1.26 (0.40, 4.43)	0.70	1.17 (0.36, 4.16)	0.80	1.14 (0.45, 2.98)	0.78	1.24 (0.48, 3.29)	0.66	1.03 (0.43, 2.55)	0.94	1.14 (0.45, 2.99)	0.79
Grit scale ^1^		1.51 (0.27, 8.85)	0.64	No adj		3.69 * (1.00, 15.00)	0.06	No adj		0.86 (0.31, 2.35)	0.78	No adj	
Participation in at least one of these: hotbed, mother roasting or steam sauna ^C^	Did not participate	—	—	—	—	—	—	—	—	—	—	—	—
	Participated in at least one	1.23 (0.19, 24.00)	0.85	1.33 (0.21, 26.00)	0.80	0.72 (0.17, 3.65)	0.66	0.76 (0.18, 3.90)	0.72	0.39 (0.08, 1.69)	0.22	0.39 (0.08, 1.69)	0.22
Confinement duration ^1,D^		1.02 (0.98, 1.06)	0.25	1.02 (0.98, 1.05)	0.42	1.01 (0.99, 1.04)	0.33	1.02 (0.46, 5.15)	0.20	0.98 * (0.96, 1.00)	0.09	0.98 (0.96, 1.01)	0.16

^1^ Continuous variables with a scale or scoring system. Variables with significant *p* value estimates are in bold * *p* < 0.1, ** *p* < 0.05. N/A—not enough observations in this group so the variable could not be modelled. CI—confidence interval. cOR—crude odds ratio. aOR—adjusted odds ratio from parsimonious regression model (including confounders using 10% statistical difference test). No Adj—using the 10% confounder rule, this model showed no significant confounders and therefore no confounder adjustments were made. ^A^—adjusted for maternal medication, number of antenatal care visits and days postpartum. ^B^—adjusted for any illness of the infant’s, number of antenatal care visits and days postpartum. ^C^—adjusted for the number of antenatal care visits. ^D^—adjusted for maternal medication, any illness of the infant, number of antenatal care visits and days postpartum.

### Appendix A.3. Unadjusted and Adjusted Cox Proportional Hazards Model

**Table A2:** Risk of exclusive breastfeeding cessation, by predictor variable and trial arm, using unadjusted and adjusted Cox proportional hazards regression models.

Variable	Category	Randomised Controlled Trial Arm											
		Control				Unconditional Social Transfer				Conditional Social Transfer			
		Unadjusted		Adjusted		Unadjusted		Adjusted		Unadjusted		Adjusted	
		cHR (95% CI)	*p*	aHR (95% CI)	*p*	cHR (95% CI)	*p*	aHR (95% CI)	*p*	cHR (95% CI)	*p*	aHR (95% CI)	*p*
Age ^A^	Younger than 25	—	—	—	—	—	—	—	—	—	—	—	—
	Between 25 and 30	0.96 (0.57, 1.59)	0.86	0.91 (0.54, 1.53)	0.73	1.03 (0.61, 1.75)	0.09	1.11 (0.64, 1.90)	0.72	1.28 (0.77, 2.14)	0.35	1.36 (0.81, 2.28)	0.25
	Older than 30	0.80 (0.49, 1.32)	0.39	0.77 (0.46, 1.28)	0.31	0.98 (0.60, 1.60)	0.93	1.02 (0.60, 1.73)	0.94	1.27 (0.72, 2.25)	0.41	1.28 (0.72, 2.28)	0.40
Marital status ^B^	Not married	—	—	—	—	—	—	—	—	—	—	—	—
	Married or cohabitating	2.12 (0.85, 5.27)	0.11	2.11 (0.85, 5.24)	0.11	0.87 (0.43, 1.73)	0.69	1.06 (0.51, 2.21)	0.88	1.60 (0.65, 3.96)	0.31	1.60 (0.65, 3.95)	0.31
Maternal Education ^A^	Primary or no education	—	—	—	—	—	—	—	—	—	—	—	—
	Secondary	1.40 (0.82, 2.41)	0.22	1.46 (0.83, 2.54)	0.19	1.39 (0.84, 2.29)	0.20	1.43 (0.85, 2.40)	0.18	0.89 (0.52, 1.50)	0.65	1.00 (0.57, 1.75)	0.99
	Tertiary	0.95 (0.55, 1.65)	0.86	0.98 (0.55, 1.77)	0.96	0.98 (0.56, 1.71)	0.94	1.04 (0.59, 1.84)	0.88	1.09 (0.66, 1.80)	0.73	1.17 (0.69, 1.97)	0.57
Employment status at six months	Not working	—	—	—	—	—	—	—	—	—	—	—	—
	Working	1.07 (0.66, 1.73)	0.78	No adj		2.19 ** (1.26, 3.95)	0.01	No adj		1.40 (0.72, 2.73)	0.32	No adj	
Household wealth ^1,A^		0.96 (0.82, 1.11)	0.56	0.95 (0.81, 1.13)	0.58	0.99 (0.85, 1.16)	0.92	1.05 (0.89, 1.24)	0.53	0.94 (0.83, 1.11)	0.62	1.00 (0.85, 1.17)	0.97
Previous births ^C^	Never given birth	—	—	—	—	—	—	—	—	—	—	—	—
	At least one previous birth	1.16 (0.76, 1.78)	0.49	1.15 (0.74, 1.77)	0.54	1.11 (0.73, 1.70)	0.62	1.28 (0.82, 2.01)	0.28	1.18 (0.78, 1.79)	0.44	1.16 (0.76, 1.76)	0.49
Intention to feed the baby	Complementary feeding	—	—	—	—	—	—	—	—	—	—	—	—
	Exclusive breastfeeding	0.88 (0.54, 1.44)	0.59	No adj		1.16 (0.58, 2.31)	0.67	No adj		0.50 * (0.23, 1.10)	0.08	No adj	
Desire for the pregnancy ^B^	Did not want the pregnancy	—	—	—	—	—	—	—	—	—	—	—	—
	Wanted the pregnancy	1.04 (0.38, 2.83)	0.95	1.03 (0.38, 2.82)	0.95	0.43 * (0.20, 0.95)	0.04	0.42 ** (0.19, 0.92)	0.03	1.28 (0.52, 3.16)	0.59	1.27 (0.51, 3.15)	0.61
Breastfeeding self-efficacy ^1^		0.94 (0.87, 1.02)	0.13	No adj		0.86 ** (0.78, 0.96)	0.00	No adj		0.99 (0.92, 1.06)	0.72	No adj	
Caregiver activities ^1^		1.10 (0.93, 1.30)	0.25	No adj		1.09 (0.96, 1.25)	0.19	No adj		1.17 * (0.98, 1.40)	0.08	No adj	
Postpartum Anxiety ^D^	Has anxiety	—	—	—	—	—	—	—	—	—	—	—	—
	No postpartum anxiety	0.88 (0.57, 1.36)	0.57	0.92 (0.59, 1.42)	0.71	1.00 (0.66, 1.52)	0.99	1.01 (0.65, 1.59)	0.95	1.40 (0.92, 2.12)	0.12	1.42 (0.93, 2.16)	0.11
Perceived stress ^E^	Low stress	—	—	—	—	—	—	—	—	—	—	—	—
	Moderate stress	0.87 (0.58, 1.32)	0.52	0.92 (0.60, 1.42)	0.71	1.09 (0.72)	0.67	1.06 (0.69, 1.61)	0.80	0.86 (0.56, 1.34)	0.52	0.81 (0.52, 1.28)	0.38
Grit scale ^1,C^		0.73 (0.38, 1.43)	0.36	0.66 (0.33, 1.31)	0.23	0.66 (0.38, 1.16)	0.15	0.62 (0.34, 1.14)	0.13	1.05 (0.64, 1.72)	0.84	1.07 (0.65, 1.75)	0.79
Participation at least one of: hotbed, mother roasting or steam sauna ^E^	Did not participate	—	—	—	—	—	—	—	—	—	—	—	—
	Participated in at least one	0.93( 0.45, 1.92)	0.85	0.93 (0.45, 1.92)	0.84	1.08 (0.54, 2.16)	0.82	1.05 (0.52, 2.10)	0.89	1.45 (0.70, 2.99)	0.32	1.33 (0.61, 2.93)	0.48
Confinement duration ^1^		0.99 * (0.98, 1.00)	0.05	No Adj		0.99 (0.98, 1.00)	0.21	No Adj		1.00 (0.99, 1.01)	0.44	No Adj	

^1^ Continuous variables with a scale or scoring system. Variables with significant *p* value estimates are in bold * *p* < 0.1, ** *p* < 0.05. N/A—not enough observations in this group so the variable could not be modelled. CI—confidence interval. cHR—crude hazard ratio. aHR—adjusted hazard ratio from parsimonious regression model (including confounders using 10% statistical difference test). No Adj—using the 10% confounder rule, this model showed no significant confounders and therefore no confounder adjustments were made. ^A^—adjusted for any illness of the infant, maternal medication and number of antenatal care visits. ^B^—adjusted for any illness of the infant. ^C^—adjusted for any illness of the infant and the number of antenatal care visits. ^D^—adjusted for maternal medication and number of antenatal care visits. ^E^—adjusted for number of antenatal care visits.
